# Poor Sleep Quality in Aging: The Association with Mental Health

**DOI:** 10.3390/ijerph20031661

**Published:** 2023-01-17

**Authors:** Ilaria Corbo, Giuseppe Forte, Francesca Favieri, Maria Casagrande

**Affiliations:** 1 Department of Psychology, Sapienza University of Rome, Via dei Marsi 78, 00185 Rome, Italy; 2 Department of Dynamic and Clinical Psychology and Health Studies, Sapienza University of Rome, 00185 Rome, Italy; 3 Body and Action Laboratory, IRCCS Santa Lucia Foundation, Via Ardeatina 306, 00179 Rome, Italy

**Keywords:** sleep, mental health, aging

## Abstract

Sleep disturbances are common in the elderly. A primary sleep disorder can result from the physiological decline of aging; however, secondary sleep problems result from various causes involving physical and mental health. Since little is known about the relationships between sleep quality and mental health in aging, the present study aims to understand how different aspects generally associated with sleep (e.g., psychological and physiological factors, and sleep medication) may predict poor sleep quality in different stages of the lifespan. Therefore, we conducted several analyses (ANOVAs, Pearson correlations, and linear regressions) to test the hypotheses of the study. Accordingly, from a pool of 180 participants (elderly, middle-aged, and young adults), 143 individuals with poor sleep quality were selected. Different predictive patterns in the three groups emerged. Specifically, the use of sleep medication associated with worse sleep conditions is predicted by poor sleep quality in the elderly and by depression in young adults. In contrast, worsening sleep quality is predicted by depression in middle-aged adults. Previous studies focused on the transitions from good to poor sleep quality, while this is the first study to have examined the features of poor sleep quality in aging, highlighting different sleep patterns across the lifespan. This evidence should be considered from a preventive perspective.

## 1. Introduction

The progressive worldwide increase of the elderly population represents a significant public health problem owing to the pathological implications frequently associated with aging [1,2,3,4].

Mental health clearly has a prominent position in the constellations of symptoms characterizing aging, owing to its role in cognitive impairment, physical, and functional decline, and in increased healthcare demand and costs [5,6].

From a mental health perspective, progressive aging is associated with worsening mood, increased anxiety, and emotional dysregulation [7,8]. Another recurring problem highlighted in studies dealing with aging is the quality of sleep. Owing to the association of sleep quality with mental and physical health [9,10,11], daily activities and quality of life [9], sleep remains an aspect that has been largely investigated as a prodrome of aging decline (for a review, see Casagrande et al., [12]).

Usually, aging is characterized by difficulty beginning and maintaining sleep (e.g., insomnia [13]) and high daytime sleepiness [12]. Accordingly, the most frequent sleep disorders reported in the lifespan are insomnia, circadian rhythm disorders, breathing-related sleep disorders (SBD), restless legs syndrome (RLS), and REM sleep behavior disorders (RBD) [13,14,15,16,17,18]. Owing to the relationships mentioned above, studies have usually focused on analyzing the interactions between mental health and sleep from a preventive perspective [19,20,21], while others have considered overt pathologies in various sleep disorders [22,23,24].

Nevertheless, sleep quality changes due to physical and psychological disorders during the lifespan are less clear. Understanding the features of this relationship in aging may help counteract poor sleep quality, which is a risk factor for age-related physiological and cognitive decline [25,26]. However, highlighting that poor sleep quality is determined by different factors in young and middle-aged people can allow the planning of specific counteracting and prevention programs for people of different ages.

Accordingly, the present study aimed to examine the association between poor sleep quality and mental health during a person’s lifespan. It is well-known that elderly and young adults generally have unhealthy sleep habits and lower sleep quality than middle-aged adults. However, changes in the characteristics of sleep quality across the lifespan (from young adulthood to late adulthood) have not been further analyzed, and no information about how sleep quality is affected by mental and physical health (e.g., blood pressure) is available. Accordingly, we aimed to understand how different aspects that generally affected sleep (i.e., mental health, use of medication, etc.) may predict poor sleep quality during the lifespan, among elderly, middle-aged, and young adults.

We hypothesized that poorer sleep quality would be differently associated with mental health (e.g., anxiety and depression) in older adults than in younger ones [11,27,28,29]. Furthermore, we posited that elderly people, middle-aged people, and young adults would report different patterns of sleep quality.

## 2. Materials and Methods

### 2.1. Participants

A total of 180 participants (age range: 19–88 years) were recruited through public notices and voluntarily took part in the study. The total sample was divided into three groups according to age, comprising 60 elderly individuals (age range: over 65; mean age: 70.3 ± 5.39; 56.7% females), 70 middle-aged adults (age range: 36–64; mean age: 53.4 ± 6.87; 50% females), and 50 young adults (age range: 18–35; mean age: 24.0 ± 4.51; 58% females).

Regarding the presence of poor sleep quality, 143 of the participants were selected on the basis of the presence of poor sleep quality (score greater than 5 on the Pittsburgh Sleep Quality Index (PSQI)), and were included in the final sample. Specifically, 48 were elderly (mean age: 70.3 ± 5.62; 62.25% females); 52 were middle-aged (mean age: 54.3 ± 6.30; 51.93% females); and 43 were young adults (mean age: 26.0 ± 4.62; 53.49% females). A history of psychiatric or psychological diagnosis, and chronic medical conditions (e.g., dementia, stroke, cardiovascular diseases, and cancer) were adopted as exclusion criteria.

### 2.2. Measures

#### 2.2.1. Socio-Demographic and Anamnestic Information

Demographic data (age, years of education, gender, and marital status), lifestyles (coffee, smoking, and alcohol consumption), medical history, and psychiatric information were collected for each patient by face-to-face interview.

#### 2.2.2. Blood Pressure

Systolic blood pressure (SBP), diastolic blood pressure (DBP), and heart rate were recorded using an automatic electronic sphygmomanometer validated for self-measurement (“personal check” PIC), according to European guidelines criteria.

The device records brachial blood pressure using the oscillometric method with a pressure range of 30–260 mmHg and a pulse rate range of 40–199 beats/min.

#### 2.2.3. Pittsburgh Sleep Quality Index

The Pittsburgh Sleep Quality Index (PSQI; [30,31]) was used to assess participants’ sleep quality over the previous 30 days through 19 questions that required information about quantitative and qualitative aspects of sleep habits.

Data were provided on seven different components of sleep: sleep quality, sleep latency, sleep duration, habitual sleep efficiency, sleep disturbance, use of sleep medication, and daytime dysfunction. A global score of sleep quality was calculated for each participant. Higher global scores indicated worse sleep quality. In line with the Italian validation, a cut-off of five was considered to define categorically good or poor sleep quality.

The overall PSQI score was calculated by adding together all the subscale scores.

For additional information, see Buysse et al. [30].

#### 2.2.4. Beck Depression Inventory

The Beck Depression Inventory (BDI; [32]) is a self-reported questionnaire including 21 items that investigate depressive symptomatology (e.g., hopelessness and irritability), cognitive (e.g., guilt and feelings of being punished), and physical symptoms (e.g., fatigue and weight loss) related to depression. Responses are given using a 5-point Likert scale. The global score indicates general depressive symptomatology. Higher scores of BDI are associated with worse conditions. Specifically, a score ranging between 0 and 13 is associated with “no depression” (i.e., no significant depressive symptoms), between 14 and 19 indicates “mild depression”, between 20 and 29 indicates “moderate depression”, and between 30 and 63 indicates “severe depression”.

#### 2.2.5. Toronto Alexithymia Scale-20

The Toronto Alexithymia Scale-20 (TAS-20; [33,34]) is a self-reported questionnaire assessing alexithymia via 20 items on a 5-point Likert scale (1 = strongly disagree, 5 = strongly agree). The test enables the assessment of three facets of alexithymia: difficulty identifying feelings (DIF), difficulty describing feelings (DDF), and externally oriented thinking (EOT). The scores range from 20 to 100 and provide both categorical and continuous information. Higher scores are associated with higher alexithymia. Scores ranging from 52 to 60 indicate moderate levels of alexithymia, and scores above 60 indicate high levels of alexithymia.

#### 2.2.6. State–Trait Anxiety Inventory

The State–Trait Anxiety Inventory (STAI; [35,36]) is a self-assessment questionnaire used to evaluate state and trait anxiety on a 4-point Likert scale (1 = not at all; 4 = very much). In line with the aim of the study, only trait anxiety (20 items) was considered. A higher STAI score identifies more elevated levels of anxiety. The questionnaire did not report a specific cut-off score for anxiety. However, conventionally, scores higher than 50.49 for men and higher than 53.10 for women indicate elevated trait anxiety.

### 2.3. Procedure

After signing an informed consent statement, the participants were subjected to blood pressure recordings (three times); then, weight and height were measured. Afterwards, the participants underwent the socio-demographic and anamnestic interview, and completed the questionnaires (Pittsburgh Sleep Quality Index, Toronto Alexithymia Scale-20, State–Trait Anxiety Inventory, and Beck Depression Inventory). The whole procedure lasted about 30 min.

### 2.4. Data Analysis

Analyses of variance (ANOVAs) were conducted to determine differences between the groups (elderly, middle-aged, and young adults) in the indices of PSQI and physical and mental health variables. To further analyze significant between-group differences, the Tukey post-hoc test was adopted. Correlations were then conducted to examine the association between the sleep indices assessed by PSQI and psychological (BDI, STAI, TAS-20) and physiological characteristics (BMI, SBP, DBP). Finally, in line with the main aim of the study, multiple regression analyses examined whether sleep quality was predicted by physiological and self-reported mental health separately in each group.

Two models were tested for each age group to analyze the possible predictors of changes in sleep quality reported by the global sleep quality index of the PSQI (model 1: physiological predictors, including BMI and blood pressure indices (SBP, DBP); model 2: psychological predictors, including STAI score, BDI score, and global TAS-20 score). Moreover, owing to the significant differences between groups in the use of medication to sleep, further multiple regressions were used to assess the predictor role of both physiological and psychological variables on medication use (model 1: physiological predictors, including BMI and blood pressure indices (SBP; DBP); model 2: psychological predictors, including STAI score, BDI score, global TAS-20 score, and total sleep quality). All statistical analyses were conducted with JASP, and a *p* < 0.05 was considered significant.

### 2.5. Ethics Statement

This study was approved by the Institutional Review Board of the Department of Psychology, Sapienza University of Rome (protocol number: 0001063). A waiver of written informed consent was granted; eligible participants signed informed consent statements before completing the questionnaire battery.

## 3. Results

Descriptive statistics for participants’ characteristics, Pittsburgh Sleep Quality Index subscales, and physiological and psychological aspects are reported in Table 1, Table 2, and Table 3, respectively.

### 3.1. Correlations

Pearson correlations between PSQI scores, psychological variables, and physiological variables are reported in Table 4 and Table 5.

### 3.2. Regression

In each group of participants, linear regressions examined the relationship of the physiological (model 1) and psychological (model 2) dimensions with the PSQI global score and the use of sleep medicine. See Table 6 and Table 7.

#### 3.2.1. Elderly

In elderly people, neither model predicted an increase in the PSQI global score (i.e., worse sleep quality). However, model 2 was marginally significant (F6,39 = 2.10; *p* = 0.08).

Regarding the use of sleep medication, model 2 was significant (F7,38 = 2.47; *p* = 0.03). In particular, high scores in global PSQI were associated with higher use of medication.

#### 3.2.2. Middle-Aged Adults

In middle-aged adults, the global PSQI score was predicted by model 2 (F6,42 = 2.78; *p* = 0.02). In particular, depressive symptoms significantly predicted higher PSQI scores. However, regarding the use of sleep medication, neither regression model was significant.

#### 3.2.3. Young Adults

In younger adults, neither model 1 nor model 2 predicted the global PSQI score. However, psychological factors predicted the use of sleep medicine (model 2) (F7,33 = 2.66; *p* = 0.03). In particular, higher depressive symptoms scores predicted higher use of sleep medication.

## 4. Discussion

The general increase in the incidence and severity of sleep disorders due to psychological, behavioral, and neurological changes associated with aging is well known [12,37,38]. Our results confirm a negative age-associated sleep quality trend [39]. Elderly people reported worse conditions than both young and middle-aged adults.

Interestingly, these findings were highlighted by considering three groups of individuals, all manifesting poor sleep quality. Although the participants showed the same severity of sleep disorders, this condition was predicted by different risk factors, highlighting a trend characterized by a different pattern across the lifespan. No physiological or psychological variable considered in this study was associated with poor sleep quality in younger and elderly people, while depression was able to predict low sleep quality in middle-aged individuals. Some of these results can be explained by the worse sleep condition of elderly people compared with young and middle-aged individuals, which allows elderly people to use more sleep medication.

Specifically, although the regression models did not highlight the role of physiological variables as predictors of sleep quality changes, higher diastolic blood pressure was associated with reduced sleep duration and efficiency in the overall sample. These results could be explained by an alteration of homeostasis via the autonomic nervous system [40], and by features of blood pressure indices (i.e., diastolic and systolic blood pressure; [41,42]), which in previous studies were related to higher night-time physiological activation [43,44]. Blood pressure directly corresponds to cardiac output, and is remarkably easy to alter, for example, by daily activity or psychological condition [1,42,45,46,47,48,49].

Moreover, another explanation regards the association between diastolic blood pressure and sleep quality as an early subclinical index of sleep disorders, such as obstructive sleep apnea [50], outlining it as a long-term predictor of sleep disorders. Anxiety and depressive symptoms are positively correlated with subjective sleep quality, sleep latency, sleep disturbance, daytime sleepiness, and global PSQI score (i.e., higher anxiety and depression are associated with worse sleep conditions). These results confirm an association between mental health and sleep quality [51,52,53].

If the general results broadly confirmed the role of psychological and physiological conditions in sleep disturbance [54], further interesting results emerged when analyses were conducted separately in the different age groups. In fact, the main aim of this work was to define possible predictors of worse sleep quality in different stages of adult life, to define risk factors of poor sleep quality in aging, and to identify specific sleep risk factors for each stage of aging. Since global sleep quality and use of medicine significantly differed between the groups, these dimensions were considered from young to elderly in order to highlight the different patterns.

In young adults showing poor sleep quality, no specific predictors of this condition were highlighted; however, depression predicted the use of sleep medication. This finding agrees with the results of other studies reporting increased use of drugs in young people with poor sleep quality, including pre-clinical conditions of sleep diseases [55,56]. Many young adults use drugs, prescribed or not, to improve sleep quality and sleep time [57]. Moreover, using medication to treat depression and other psychiatric conditions in young adults may result in disrupted sleep, which can explain why this association was reported in individuals with poor sleep quality [58].

However, further studies should investigate this association by using polysomnographic recordings and more extensive psychological assessment. A more comprehensive evaluation could differentiate between different clinical conditions associated with poor sleep quality, such as sleep disorders and psychopathologies.

For middle-aged adults, depression appears to be associated with worse sleep quality. These results are in line with previous research data. Middle-aged and older adults with major depression demonstrated agitation and somatic symptoms more than younger people with major depression (for a meta-analysis, see Hegeman et al., [59]).

The rate of sleep disturbance in depression ranges from 10–40% in middle-aged adults, with a lower rate in late adulthood than in early adulthood [60]. Moreover, it has been reported that mental health conditions influence middle-aged and older adults in their sleep architecture. In particular, depressive symptoms have been shown to be the main risk factors for sleep disturbance and low sleep quality [61]. Our study confirms this association between poor sleep quality and depressive symptomatology, starting in middle-age, although no conclamant depressive disorder was reported.

Considering elderly people, a general alteration in mental health predicts worse sleep quality. Moreover, higher use of sleep medication is associated with poorer sleep quality.

In summary, to answer the questions raised in this research, some psychological variables play a greater role than others in predicting sleep disturbances. From a mental health point of view, anxiety appears to have a moderate role in predicting sleep alteration in a population of elderly people who already exhibit poor sleep quality, as suggested by prior studies that reported an association between anxiety and sleep disturbances [62]. However, the evidence generally suggests that there is a close interplay between mental health (i.e., anxiety/depression [63]), sleep quality and sleep disorders (i.e., insomnia), and health-related quality of life in older adults [64,65]. In this sense, the significant association that emerged in the second regression model, i.e., mental health conditions and the use of sleep medication, can highlight the different patterns of sleep quality in young and elderly people. While in elderly people the use of sleeping drugs is associated significantly with effective poor sleep quality assessed by the global quality sleep index, in young adults depression is the main mental health dimension associated with the use of sleeping drugs [51,62,63]. In conclusion, in young individuals, attempts to alleviate mental health conditions can allow the development of other symptoms, such as sleep disorders. Conversely, the worse sleep pattern characterizing elderly people can justify their use of sleep medication. Further studies should investigate the relationship between sleep disorders and drug use in aging with polysomnographic methods and from a longitudinal perspective.

### Limitations

One of the main limitations of this study is its cross-sectional nature, which does not allow for establishing the direction of causality between aging and physiological and psychological conditions, as well as their connections. Another limitation of the study is the self-reported nature of sleep conditions. While this method of defining sleep quality is supported by the literature, our study cannot define a sleep disorder diagnosis in the participants, reducing the generalizability of the evidence, since this can be associated only with altered sleep quality rather than with diagnosed sleep disorders. Accordingly, further studies should overcome these limitations by using polysomnographic sleep recordings.

A further limitation can be identified in the study’s consideration of a restricted frame of mental health. Other dimensions, such as mental distress, anger, personality traits (e.g., impulsivity), and cognitive traits (e.g., executive functioning) associated with altered homeostasis and the physiology of sleep, should be included in future studies [66,67].

## 5. Conclusions

People diagnosed with mental illness are more likely to have poor sleep quality than the general population. Starting from this assumption, this study expands this notion, underlining that some physical and mental variables can affect sleep quality in people with poor sleep quality. These conditions change between younger, middle-aged, and elderly people. To counteract this pattern, it could be useful to plan specific interventions to improve sleep in young, middle-aged, and elderly people; for example, it might be useful to improve mood in the middle-aged and young groups, and sleep quality in the elderly. In fact, the presence of poor subjective sleep quality could be a prodrome of a diagnosable sleep disorder, and could indicate a worsening of the general psychological condition of the individual. Most previous studies had focused on the first part of the continuum, i.e., the transition from good to poor sleep quality. In contrast, as far as we know, no studies had investigated the phenomena that characterize established poor sleep quality.

An important consequence of poor mental health and sleep quality is an increased demand for health services, with associated socioeconomic costs. For these reasons, it is relevant to consider this issue. Further studies should confirm and extend the results of this study, also using polysomnographic recordings to assess the quality of sleep in order to plan diversified interventions to enhance sleep quality according to the individual’s age. Improving sleep quality would entail a better general state of physical, psychological, and cognitive health, with evident beneficial effects on aging.

## Figures and Tables

**Table 1:** Characteristics of participants.

	Elderly	Middle Aged Adults	Young Adults
Age—mean (SD)	70.3 (5.62)	54.3 (6.30)	26.0 (4.62)
Education—mean (SD)	14.6 (4.15)	16.0 (3.97)	17.1 (2.85)
Female—n° (%)	30 (62.25)	27 (51.93)	23 (53.49)
Marital Status			
Single/Divorced—n° (%)	12 (25)	15 (28.85)	42 (97.67)
Married/Cohabiting—n° (%)	29 (60.42)	36 (69.23)	1 (2.33)
Widowed—n° (%)	7 (14.58)	1 (1.92)	0 (0)
Lifestyles Habits			
Coffee—n° (%)	42 (87.5)	46 (88.46)	36 (83.72)
Smoker—n° (%)	10 (20.83)	11 (21.15)	18 (41.86)
Alcohol user—n° (%)	30 (62.5)	36 (69.23)	33 (76.74)

**SD**: Standard Deviation.

**Table 2:** Between-group comparisons of sleep characteristics (Pittsburgh Sleep Quality Index).

	Elderly	Middle Aged Adults	Young Adults	F	p	η^2^p
Subjective Sleep Quality	1.21 (0.58)	1.33 (0.62)	1.07 (0.70)	1.94	0.15	0.03
Sleep Latency	1.04 (0.90)	1.10 (1.03)	1.07 (1.01)	0.04	0.96	0.01
Sleep Duration	1.27 (0.74)	1.06 (0.73)	0.98 (0.77)	1.94	0.15	0.03
Habitual Sleep Efficiency	2.92 (0.35)	2.87 (0.40)	2.86 (0.47)	0.28	0.76	0.01
Sleep Disturbance	1.29 (0.50)	1.33 (0.59)	1.16 (0.53)	1.16	0.32	0.02
Use of Sleep Medication	0.92 (1.27)	0.21 (0.64)	0.05 (0.31)	14.00	<0.01 ab	0.20
Daytime Dysfunction	0.65 (0.60)	0.75 (0.68)	0.79 (0.71)	0.59	0.56	0.01
Global PSQI Score	9.29 (2.46)	8.64 (2.33)	7.98 (2.13)	3.66	0.03 a	0.05

F and p refer to ANOVA comparisons. Significance: *p* < 0.05. PSQI: Pittsburgh Sleep Quality Index; a: significant difference between elderly and young adults; b: significant difference between elderly and middle-aged adults.

**Table 3:** Between-group comparisons of physiological and psychological variables.

	Elderly	Middle Aged Adults	Young Adults	F	*p*	η^2^p
Body Mass Index	25.90 (3.47)	25.50 (4.68)	23.40 (4.40)	4.64	0.01 ac	0.06
Systolic Blood Pressure	131.40 (20.25)	121.70 (16.26)	112.80 (14.85)	12.90	<0.01 abc	0.16
Diastolic Blood Pressure	76.80 (9.83)	77.20 (9.91)	71.30 (9.35)	5.12	0.01 ac	0.07
BDI	8.30 (8.28)	5.42 (4.15)	7.11 (6.18)	2.55	0.08	0.04
STAI	39.13 (10.16)	36.13 (8.30)	41.40 (10.80)	3.39	0.04 c	0.05
TAS-20	45.83 (14.20)	38.99 (10.98)	41.12 (13.00)	3.71	0.03 b	0.05

F and p refer to ANOVA comparison. Significance: *p* < 0.05. a: significant difference between elderly and young adults; b: significant difference between elderly and middle-aged adults; c: significant differences between middle-aged adults and young adults; BDI: Beck Depression Inventory; STAI: State–Trait Anxiety Inventory; TAS-20: Toronto Alexithymia Scale-20.

**Table 4:** Correlations between Pittsburgh Sleep Quality Index and physiological variables.

		Sleep Quality	Sleep Latency	Sleep Duration	Habitual Sleep Efficiency	Sleep Disturbance	Use of Sleep Medicine	Daytime Dysfunction	Global PSQI Score
Body Mass Index	r p	0.06 0.50	0.06 0.48	0.03 0.76	0.00 1.00	0.10 0.24	0.04 0.60	−0.01 0.87	0.01 0.32
Systolic Blood Pressure	r p	−0.03 0.75	−0.05 0.60	0.06 0.51	0.09 0.31	0.05 0.57	0.09 0.32	−0.08 0.37	0.03 0.72
Diastolic Blood Pressure	r p	−0.03 0.72	−0.03 0.75	0.14 0.10	0.17 0.05 *	−0.07 0.40	−0.09 0.27	−0.12 0.18	−0.33 0.70

*: *p* < 0.05.

**Table 5:** Correlations between Pittsburgh Sleep Quality Index and psychological variables.

		*Sleep Quality*	*Sleep Latency*	*Sleep Duration*	*Habitual Sleep Efficiency*	*Sleep Disturbance*	*Use of Sleep Medicine*	*Daytime Dysfunction*	*Global PSQI Score*
*BDI*	*r* *p*	*0.33* <0.01 *	*0.28* <0.01 *	*0.15* *0.08*	*0.03* *0.72*	*0.26* 0.01 *	*0.11* *0.20*	*0.16* *0.05*	*0.07* *0.40*
*STAI*	*r* *p*	*0.21* 0.01 *	*0.28* <0.01 *	*0.08* *0.33*	*−0.03* *0.75*	*0.14* *0.09*	*0.09* *0.30*	*0.15* *0.08*	*0.30* <0.01 *
*TAS-20*	*r* *p*	*0.15* *0.09*	*−0.07* *0.41*	*0.06* *0.49*	*−0.04* *0.62*	*0.08* *0.37*	*−0.09* *0.28*	*0.07* *0.40*	*0.03* *0.77*

*: p < 0.05; BDI: Beck Depression Inventory; STAI: State–Trait Anxiety Inventory; TAS-20: Toronto Alexithymia Scale-20. Note: higher PSQI scores indicate worse sleep characteristics

**Table 6:** Linear regression on Pittsburgh Sleep Quality Index.

Global PSQI Score	Elderly		Middle Aged		Young Adults	
	B	SE (B)	B	SE (B)	B	SE (B)
Model 1						
BMI	0.01	0.11	−0.11	0.08	0.17	0.08
Systolic Blood Pressure	0.01	0.02	−0.05	0.04	−0.02	0.03
Diastolic Blood Pressure	−0.01	0.05	0.05	0.07	−0.03	0.04
R	0.07		0.27		0.37	
R^2^	0.01		0.07		0.14	
p	0.97		0.33		0.14	
Model 2						
BMI	−0.06	0.10	−0.05	0.07	0.15	0.08
Systolic Blood Pressure	0.03	0.02	−0.04	0.04	0.01	0.03
Diastolic Blood Pressure	−0.04	0.04	0.04	0.06	−0.05	0.05
BDI	0.08	0.06	0.25 *	0.09	0.15	0.09
STAI	0.08	0.04	0.01	0.05	0.01	0.04
TAS-20	−0.04	0.03	−0.02	0.03	−0.04	0.03
R	0.50		0.53		0.50	
R^2^	0.24		0.28		0.25	
p	0.08		0.02 *		0.11	

BDI: Beck Depression Inventory; STAI: State–Trait Anxiety Inventory; TAS-20: Toronto Alexithymia Scale-20; *: *p* < 0.05; SE: Standard Error.

**Table 7:** Linear regression on use of sleep medication.

Use Of Sleep Medication	Elderly		Middle Aged		Young Adults	
	B	SE (B)	B	SE (B)	B	SE (B)
Model 1						
BMI	−0.03	0.06	−0.02	0.02	0.01	0.01
Systolic Blood Pressure	0.01	0.01	−0.02	0.01	−0.00	0.01
Diastolic Blood Pressure	−0.04	0.03	−0.01	0.02	−0.00	0.01
R	0.25		0.20		0.11	
R^2^	0.06		0.034		0.01	
p	0.44		0.62		0.93	
Model 2						
BMI	−0.04	0.05	−0.02	0.02	−0.01	0.01
Systolic Blood Pressure	0.01	0.01	0.01	0.01	0.01	0.01
Diastolic Blood Pressure	−0.03	0.02	−0.01	0.02	−0.01	0.01
BDI	0.01	0.03	−0.03	0.03	0.04 *	0.01
STAI	0.01	0.02	−0.01	0.01	−0.01	0.01
TAS-20	−0.02	0.01	−0.01	0.01	−0.01	0.01
Global PSQI Score	0.20 *	0.08	0.11 *	0.05	0.01	0.02
R	0.56		0.41		0.60	
R^2^	0.31		0.20		0.36	
p	0.03 *		0.34		0.03 *	

BDI: Beck Depression Inventory; STAI: State–Trait Anxiety Inventory; TAS-20: Toronto Alexithymia Scale-20; *: *p* < 0.05; SE: Standard Error.

## Data Availability

Data are available. Please contact corresponding authors.
